# Preserving Privacy of Internet of Things Network with Certificateless Ring Signature

**DOI:** 10.3390/s25051321

**Published:** 2025-02-21

**Authors:** Yang Zhang, Pengxiao Duan, Chaoyang Li, Hua Zhang, Haseeb Ahmad

**Affiliations:** 1 College of Food and Bioengineering, Zhengzhou University of Light Industry, Zhengzhou 450001, China; 2 College of Software Engineering, Zhengzhou University of Light Industry, Zhengzhou 450001, China; 3 Department of Computer Science, National Textile University, Faisalabad 37610, Pakistan

**Keywords:** certificateless, IoT network, lattice cryptography, post-quantum, ring signature

## Abstract

With the rapid development of quantum computers and quantum computing, Internet of Things (IoT) networks equipped with traditional cryptographic algorithms have become very weak against quantum attacks. This paper focuses on the privacy-preserving problem in IoT networks and proposes a certificateless ring signature (CLRS) scheme. This CLRS is constructed with lattice theories, which show promising advantages in resisting quantum attacks. Meanwhile, the certificateless mechanism reduces the key control ability of the key generation center (KGC) by adding personal secret keys to the private key generated by the system. Meanwhile, the ring signature mechanism protects users’ privacy information through a non-central control mechanism. Next, the security proof in a random oracle model is given, which shows that this CLRS scheme can obtain unforgeability and ensure the signer’s anonymity. Its security properties include non-repudiation, traceability, and post-quantum security. Then, the efficiency comparison and performance results show that this CLRS scheme is more efficient and practical than similar schemes. Moreover, this work presents an exploration of the post-quantum cryptographic algorithm and its application in IoT networks.

## 1. Introduction

IoT network security is a critical aspect of modern technology infrastructure, as it safeguards the vast array of interconnected devices from potential cyberattacks, data breaches, and unauthorized access. As the Internet of Things (IoT) continues to expand across industries such as healthcare, manufacturing, smart cities, and home automation, the sheer volume of connected devices creates a broad attack surface that malicious actors can exploit [1]. Each device, whether it is a smart thermostat, industrial sensor, or wearable health monitor, acts as a potential entry point for cybercriminals, making robust security measures essential. One of the biggest challenges in IoT network security is the limited computing power of many IoT devices, making it difficult to implement traditional security protocols such as encryption and authentication, especially when faced with quantum attacks [2]. As a result, attackers can exploit weak or outdated security features, such as default passwords or unpatched firmware, to gain access to the network.

Privacy preservation in IoT networks is a paramount concern, as the proliferation of interconnected devices continuously generates vast amounts of sensitive data [3], such as personal health information, location data, and user preferences, which, if compromised, can lead to severe privacy violations and identity theft. With IoT devices embedded in everyday life, from smart home appliances to wearable health trackers, users’ private information is often transmitted across a network, raising significant concerns about unauthorized access, surveillance, and misuse. The decentralized and heterogeneous nature of IoT networks further complicates privacy protection, as IoT devices, often with limited processing power, may not have the capacity to handle robust security measures. To address these challenges, privacy-preserving techniques must ensure that sensitive information is protected from unauthorized access while enabling IoT networks’ functionality and convenience.  Advanced encryption algorithms and secure communication protocols can help ensure that data remain private during transmission, preventing interception by malicious actors [4]. In an IoT environment, cryptography is used to protect sensitive data, such as personal information, device status, and environmental data, by encrypting them during transmission and ensuring that unauthorized parties cannot intercept or alter them. Symmetric and asymmetric encryption techniques are employed to maintain confidentiality, while hashing algorithms are used to verify data integrity. However, the use of cryptography alone is not sufficient to guarantee the authenticity of devices and the integrity of data within an IoT network. This is where digital signatures come into play. A digital signature, which involves the use of private and public keys, ensures that data come from a legitimate source and have not been tampered with during transmission [5]. When a device sends data to another device or to a server, a digital signature can be applied to verify the identity of the sender and validate the message’s integrity. This process helps mitigate risks such as person-in-the-middle attacks, device impersonation, and data manipulation, which are common in IoT ecosystems. As IoT devices are often resource-constrained, implementing lightweight cryptographic protocols that balance security with energy efficiency is essential for maintaining the security of the network without overwhelming the devices.

Post-quantum cryptographic algorithms are designed to secure data and communications against the potential threats posed by quantum computers, which have the ability to break widely used encryption methods like RSA and ECC (elliptic curve cryptography) by efficiently solving problems that are currently considered computationally infeasible for classical computers [6]. As quantum computing continues to evolve, the development of cryptographic systems resistant to quantum attacks is crucial for maintaining the confidentiality, integrity, and authenticity of digital information in a future where quantum machines are commonplace [7,8,9,10]. These post-quantum algorithms aim to provide secure alternatives by relying on mathematical problems that are believed to be hard for quantum computers to solve, such as lattice-based, code-based, hash-based, and multivariate polynomial systems. One of the primary goals of post-quantum cryptography is to ensure that, even as quantum technologies advance, critical infrastructure such as financial systems, healthcare databases, and national security communications remain protected from potential vulnerabilities. The process of standardizing these new algorithms, led by institutions like the National Institute of Standards and Technology (NIST), is currently underway, intending to create secure cryptographic protocols that can be seamlessly integrated into existing systems. Lattice-based cryptography, a promising post-quantum cryptographic technique, provides robust security for IoT networks by offering resistance against quantum computing attacks, which threaten to break traditional encryption methods [11]. In the context of the IoT, where a multitude of interconnected devices often transmit sensitive data, lattice-based digital signatures ensure both data integrity and authenticity. These signatures rely on mathematical problems derived from lattice structures, which are considered difficult for quantum computers to solve, making them a secure alternative to conventional digital signature algorithms such as RSA and ECDSA. Lattice-based cryptography allows for smaller key sizes and more efficient performance compared to other post-quantum algorithms, making it a practical choice for resource-constrained IoT devices. This helps ensure that even as quantum technologies evolve, IoT devices can securely authenticate communications, protect data integrity, and prevent malicious activities such as impersonation or data tampering, fostering a trust-based environment within increasingly complex and distributed IoT ecosystems.

This paper proposes a lattice-based certificateless ring signature (CLRS) scheme to protect the privacy of IoT networks.

1. This paper first proposes a CLRS scheme based on a lattice assumption. This CLRS scheme utilizes the bimodal Gaussian distribution to improve the key generation efficiency, a certificateless mechanism to weaken the centralized risk of KGC, and  a ring signature mechanism to achieve unconditional anonymity.
2. This paper presents a formal security proof of the proposed CLRS scheme in a random oracle model. The  results show that this CLRS achieves unforgeability and anonymity. Meanwhile, an additional analysis also shows that it features non-repudiation, traceability, and anti-quantum security.
3. This paper provides a comparative analysis and performance evaluation of the proposed CLRS scheme. The results show that this CLRS scheme is more efficient and practical than related schemes in strengthening IoT network security.

In the following, related works are presented in Section 1, some preliminaries are presented in Section 2, the proposed CLRS scheme is presented in Section 3, the security proof is presented in Section 4, its performance is presented in Section 5, and a conclusion is presented in Section 6.

## 2. Related Work

### 2.1. Privacy-Preserving in IoT Network

In current IoT networks, the massive number of interactions between IoT devices and high-frequency data create many privacy-preserving problems, which threaten the privacy and security of sensitive data and users. Meanwhile, many privacy-preserving methods have been proposed in recent years that are data-oriented or user-oriented. Kumar et al. [12] designed a privacy-preserving framework for IoT-based smart cities, which utilizes blockchain to achieve distributed IoT data sharing and the principal component analysis technique to transform the form of the data. That method is based on traditional cryptographic algorithms, which cannot resist quantum attacks. Ruzafa et al. [13] proposed a federated-learning-based intrusion detection method for industrial IoT, and they also utilized differential privacy techniques to protect IoT data privacy. Ye et al. [14] introduced a trust evaluation mechanism and proof-of-trust protocol to establish a scalable blockchain-based IoT network, and they also proposed a smart-contract-based privacy protection scheme to improve IoT data security. These authors did not present a new cryptographic algorithm to improve privacy security. Yin et al. [15] presented a distributed identity with blockchain technology to construct a self-sovereign identity, which is very suitable for solving IoT identity problems and strengthening the ownership of personal user data. Das et al. [3] established a lightweight authentication mechanism for IoT device security and utilized unclonable functions to save IoT device storage and processing power. This hash-based method can improve algorithm efficiency, but it is still weak against quantum attacks.

Although there are many privacy-preserving methods, the cryptography algorithm is a more basic and robust method of guaranteeing the security of IoT data and system users. The cryptography algorithm uses this difficult mathematical problem as the foundation and constructs a safety barrier for information systems. This paper designs a new signature scheme for IoT networks.

### 2.2. Post-Quantum Cryptography for IoT Network

When facing quantum attacks,  PQC algorithms can support a system’s security in IoT networks. For these PQC algorithms, lattice cryptography and hash are two common designs. Yi [7] constructed a post-quantum blockchain system for social IoT and proposed a ring signature to protect data privacy. This post-quantum blockchain suffers from the centralized management problem. Cohen et al. [16] created a coding scheme that applies an arbitrary secure cryptosystem and a pre-processing operation to achieve individual and anti-quantum security. This code-based scheme is inefficient with a big key size. Señor et al. [17] evaluated a standard post-quantum algorithm selected by the NIST and utilized the Contiki-NG operating system to apply it in wireless sensor networks. Xu et al. [18] designed a nested hash access system to protect the initial access of massive IoT devices, who also applied post-quantum encryption to improve the security against malicious quantum adversaries. Comparing these four PQC schemes,  code-based algorithms are suitable for encryption schemes,  hash-based algorithms are suitable for the digital signature scheme but generally as part of the scheme’s steps, and  multivariate-based algorithms are not suitable for IoT network with big keys. Lattice-based methods are considered to be the most promising PQC technology because of their high computing speed and low communication overhead.

PQC has gained considerable attention in recent years, especially in finance and government. As the IoT has penetrated every aspect of people’s lives, anti-quantum privacy preservation is becoming increasingly essential. This paper designs a CLRS scheme based on lattice cryptography to improve the system security of IoT networks.

### 2.3. Lattice-Based Signature for IoT Network

Lattice cryptography has the advantages of high security, flexibility, and high encryption/decryption, so applying this theory to construct PQC schemes for IoT networks is promising. Yavuz et al. [5] introduced a multiple-time ECC signature scheme for resource-constrained IoT devices, and they achieved a small key size by reducing the scalar multiplication or addition operations. But, this ECC-based scheme cannot resist quantum attacks. Zhang et al. [8] established a three-layered security model to protect the data transmitted in IoT networks and proposed an identity-based signature based on lattice theory to improve post-quantum security. This scheme utilizes an identity mechanism to simplify key management, but it is not efficient. Roy et al. [19] proposed an authentication protocol for a three-layered IoT network composed of cloud, fog, and edge devices, and they applied post-quantum theory to achieve proper access to IoT devices. Li et al. [10] constructed a cross-chain health-data-sharing platform and introduced a designated verifier signature scheme to protect the privacy of cross-chain transactions among different Internet of Medical Things (IoMT) systems. This scheme experiences the key escrow problem as the keys are managed by KGC. Bouakkaz et al. [20] utilized lattice theory to design a certificateless signature scheme and applied it to vehicular ad hoc networks to protect the privacy of system users and data. Dong et al. [21] proposed a CLRS scheme with a lattice assumption and utilized the bimodal Gaussian distribution to improve the sampling probability. The secret key size and time consumption of the signing process of this CLRS scheme are large.

These lattice signature schemes have certain advantages in IoT applications, but they also suffer from storage space limitations and efficiency problems with storage- and resource-constrained IoT devices. This work designs a more efficient CLRS scheme with lattice assumptions to cover these problems in most IoT networks.

## 3. Preliminaries

### 3.1. Lattice Theory

Some parameters are pre-defined in Table 1.

**Definition** **1** (*Lattice* [22])**.** *$v_{1},\ldots ,v_{n}\in R^{m}$ is an independent vector; $\Lambda_{L}$ in Equation (1) represents the lattice:*

(1) $$\Lambda_{L}=\left\{a_{1}v_{1}+a_{2}v_{2}+\ldots +a_{n}v_{n}:a_{1},a_{2},\ldots ,a_{n}\in Z\right\}$$

*Here, $A=\left(a_{1},\ldots ,a_{m}\right)\subset R^{n\times m}$ is the coefficient matrix of $\Lambda_{L}$ with dimensions n and rank m, where m=O(nlogq).*

**Definition** **2** (*q-ary Lattice* [22])**.** *The “q-ary” lattice is a dual lattice of $\Lambda_{L}$, which is defined with a prime number q and matrix $A\in Z_{q}^{n\times m}$, as shown in Equation (2).*

(2) $$\begin{array}{cc} \; & L^{⊥}\left(A\right)=\left\{x\in Z^{m}|Ax=0\;modq\right\}\\ \; & L_{y}^{⊥}\left(A\right)=\left\{x\in Z^{m}|A^{T}y=x\;mod\;q\;for\;y\in Z^{n}\right\} \end{array}$$

**Definition** **3** (*Trapdoor algorithm* [23])**.** *For any prime q=poly(n) and any m≠5nlogq, there is a probabilistic polynomial-time algorithm that, on input $1^{n}$, outputs a matrix $A\in Z_{q}^{n\times m}$ and a full-rank set $S\subset L^{⊥}\left(A\right)$, where the distribution of A is statistically close to uniform over $Z^{n\times m}$, and the length $\left||S|\right|\leq L=m^{2.5}$.*

**Definition** **4** (*Gaussian distribution* [24])**.** *With standard deviation σ∈R and center c∈R evaluated at x∈R, the Gaussian distribution is $\rho_{c,\sigma }\left(x\right)=exp\left(\frac{-{\left(x-c\right)}^{2}}{2\sigma^{2}}\right)$, and $\rho_{c,\sigma }\left(x\right)=exp\left(\frac{-||x-{c||}^{2}}{2\sigma^{2}}\right)$ (general version) for $x,c\in R^{n}$. Here, $\rho_{\sigma }\left(x\right)$ is a Gaussian distribution with c=0. $D_{\sigma }\left(x\right)=\rho_{\sigma }\left(x\right)/\rho_{\sigma }\left(Z\right)$ is the discrete Gaussian distribution over Z with c=0. $D_{\sigma }\left(x\right)=\rho_{\sigma }\left(x\right)/\rho_{\sigma }\left(Z^{m}\right)$ is the more general situation over $Z^{m}$ with c=0.*

**Definition** **5** (*$ℜ-SIS_{q,n,m,\beta }^{\kappa }$ problem* [24])**.** Givne ring ℜ and a distribution κ over ${ℜ}_{q}^{n*m}$, $ℜ-SIS_{q,n,m,\beta }^{\kappa }$ is a problem where a non-zero $v\in {ℜ}_{q}^{m}$ must be found for Equation (3):

(3) Av=0modq

where $A\in {ℜ}_{q}^{n*m}$, and ${\left||v|\right|}_{2}\leq \beta$.

### 3.2. Model Definitions

This section presents the scheme’s model and the security model.

(1) Scheme’s model

The CLRS scheme is composed of five algorithms: Setup, Partial KeyGen., KeyExt, Sign, and Verify. These algorithms are all executed in probabilistic polynomial time.

- **Setup ($1^{n}$):** Initiate a security parameter *n*; KGC generates the system parameters pp.
- **Partial KeyGen. (pp):** KGC utilizes the pp to generate the partial public and secret key pair (ppk,psk) for a new user.
- **KeyExt. (pp,ppk,psk):** User utilizes pp, ppk, and psk to derive their public and secret key pair (pk,sk).
- **Sign (pp,pk,sk,μ):** User utilizes pp, pk, and sk to sign the message μ and output a signature $s_{\mu }$.
- **Verify ($\mathrm{pp},\mathrm{pk},\mu ,s_{\mu }$):** Verifier utilizes pp, pk, and μ to verify the validity of signature $s_{\mu }$, Then, outputs are accepted or rejected.

(2) Security model

A query–respond game was established in a random oracle to prove the unforgeability and anonymity of this CLRS scheme. Two participants, adversary Eve *E* and challenger *C*, execute this game to achieve personal goals. With a reasonable assumption, the following two definitions utilized this game to prove the unforgeability and anonymity of this method of proof via contradiction.

**Definition** **6** (Unforgeability)**.** *Under a proper hypothesis of E, C can utilize the query results and a forged signature to solve an SIS instance.*

- *Initialize*: *C* initiates the system parameters pp.
- *Query*: *E* performs the following queries with *C* and tries to obtain enough information to increase the probability of forging a legitimate signature.
  – *Partial secret key query*: *E* queries the non-target user *i* about the partial private key $psk_{i}$.
  – *Secret key query*: *E* queries the non-target user *i* about the private key $sk_{i}$.
  – *H query*: *E* queries the non-target message μ about the hash algorithm *H*.
  – *Signature query*: *E* queries the non-target message μ about its signature $s_{\mu }$
- *Forge*: *E* utilizes the information obtained to forge a signature $s_{\mu^{*}}$ of the target message $\mu^{*}$.
- *Challenge*: *C* also can generate a signature $s_{\mu^{**}}$ of the target message $\mu^{*}$ via the forking lemma [25]. Then, *C* attempts to utilize these two signatures, $s_{\mu^{*}}$ and $s_{\mu^{**}}$, to solve the SIS instance.
- *Analyze*: Analyze whether the SIS problem can be solved or not. Meanwhile, the successful forgery probability can be computed, and the security-proof results are confirmed.

**Definition** **7** (Anonymity)**.** *Under the hardness of the SIS problem, E cannot distinguish the real signer from between two different users in relation to two different signatures.*

- *Initialize*: *C* initiates the system parameters pp.
- *Query*: *E* performs the queries with *C* about the partial secret key, secret key, *H*, and signature.
- *User creation*: *E* creates two different users, i=0 and i=1.
- *Signature construction*: *C* generates a signature $s_{\mu^{*}}^{i}$ for the target message $\mu^{*}$ by randomly selecting one user: i=0 or i=1.
- *Guess*: With the former query results, *E* guesses $s_{\mu^{*}}^{i}$ to determine whether the signature $s_{\mu^{*}}^{i}$ is generated by user i=0 or i=1.
- *Analyze*: With enough guess results, *C* analyzes the advantages of and probability that *E* can make the correct guess.

## 4. The Proposed CLRS

$ℜ-SIS_{q,n,m,\beta }^{\kappa }$ represents the advantage of an anti-quantum attack and whether this CLRS scheme can protect IoT network security in the quantum computer age with this lattice assumption. The key generation center (KGC) initializes the security parameter *n*, other system parameters q,m, and a hash function $H:\;{\left\{0,1\right\}}^{*}\to Z_{q}^{n}$. Here, *q* serves as a prime number that satisfies q=ploy(n)≥3, and *m* serves as a positive integer that satisfies m≥5nlogq. The system public parameter is pp=(q,n,m,H). After these setup processes, the following four algorithms, ParitalKeyGen., KeyExt., Sign, and Verify, constitute this CLRS scheme.

PartialKeyGen.$\;(1^{n})$: For every new IoT network user, KGC executes Algorithm 1 to generate a partial key pair $\left(ppk_{i},psk_{i}\right)$ and returns it to the user. The detailed steps are given below.
**Algorithm 1** Partial KeyGen.**Input:** *q*, *n*, and *m***Output:** Ring user partial key pair $\left(ppk_{i},psk_{i}\right)$;1:KGC executes the $TrapGen(1^{n})$ algorithm to generate a matrix $T_{i}\in Z_{2q}^{n\times m}$;2:Generate a matrix $S_{i}\in Z_{2q}^{m\times n}$, which satisfies $T_{i}S_{i}=T_{i}\left(-S_{i}\right)=qI_{n}\;mod\;2q$;3:Serve $\left(T_{i},S_{i}\right)$ as $\left(ppk_{i},psk_{i}\right)$ and send it to ring user *i*.

KeyExt.$\;(pp,ppk_{i},psk_{i})$: The ring user executes Algorithm 2 to derive their own public/private key pair $\left(pk_{i},sk_{i}\right)$. Here, the user selects their own secret information and composes it with partial public and private keys. The detailed steps are given below.
**Algorithm 2** KeyExt.**Input:** $\left(ppk_{i},psk_{i}\right)$**Output:** Key pair $\left(pk_{i},sk_{i}\right)$1:Ring user *i* randomly selects a matrix $K_{i}\in Z_{2q}^{m\times m}$;2:Computes their public key $A_{i}=T_{i}K_{i}$;3:Computes their private key $B_{i}=K_{i}^{-1}S_{i}$;4:Serves key pair $\left(A_{i},B_{i}\right)$ as $\left(pk_{i},sk_{i}\right)$ and opens $pk_{i}=A_{i}$.

Sign$\;(pp,pk_{i},sk_{i},\mu )$: A ring user $\left(pk_{k},sk_{k}\right)=\left(A_{k},B_{k}\right)$, i=k executes this Algorithm 3 to sign a message μ on behalf of the ring $R=\left\{pk_{1},pk_{2},\ldots ,pk_{l}\right\}=\left\{A_{1},A_{2},\ldots ,A_{l}\right\}$. Detailed steps are given below.
**Algorithm 3** Sign**Input:** μ, $\left(A_{k},B_{k}\right)$, $R=\{A_{1},A_{2},\ldots ,A_{l}\}$**Output:** CLRS s(μ)1:Ring user *k* randomly selects $y_{1},y_{2}\in D_{\sigma }^{m}$;2:Computes $x=\sum_{i=1}^{l}A_{i}y_{2}$;3:Computes $z=H(A_{k}y_{1}+x\;mod\;2q,\mu )$;4:Randomly selects t∈{0,1};5:Computes $e=y_{1}+{\left(-1\right)}^{t}B_{k}z$ with probability $min(\frac{D_{\sigma_{2}}^{m}\left(y_{2},y_{3}\right)}{MD_{v_{2}\sigma_{2}}^{m}\left(y_{2},y_{3}\right)},1)$; otherwise, restarts;6:Serves (e,z,x) as the CLRS s(μ).

Verify$\;(pp,\mu ,pk_{k},s\left(\mu \right))$: The verifier executes Algorithm 4 to check the legality of this CLRS <e,z,x>. The detailed steps are given below.
**Algorithm 4** Verify**Input:** μ, $A_{k}$ and (e,z,x)**Output:** Reject or accept1:**if** ||e||>L **then**2:   Reject it3:**end if**4:**if** ${\left||e|\right|}_{\infty }\geq q/4$ **then**5:   Reject it6:**end if**7:When $z=H(A_{k}e+x+qz\;mod\;2q,\mu )$ holds, accept; otherwise, reject.

Note that in the signing step, *M* is a fixed positive real number, which can guarantee a 100% preceding probability of signature generation. In the verification step, *L* is an acceptance bound that satisfies $L=\eta \sqrt{m}\sigma$, where η∈[1.1,1.4]. This CLRS scheme can guarantee the accuracy, completeness, and verifiability of the IoT data in the data-sharing process. Meanwhile, the certificateless mechanism can weaken the KGC and reduce the risk of privacy breaches due to a malicious KGC. Moreover, this ring signature mechanism can achieve anonymity and protect the personal privacy of real signers.

## 5. Security Analysis

A secure CLRS scheme should pass a formal security proof in the random oracle model. This section first provides the correctness analysis, proves the unforgeability and anonymity of the scheme, and then gives other related security analyses.

### 5.1. Correctness

First and foremost, signature *e* in CLRS <e,z,x> should satisfy ||e||≤L and ${\left||e|\right|}_{\infty }<q/4$. If it passes this verification, the verifier continues to verify the following condition shown in Equation (4).

(4) $$H\left(A_{k}e+x+qz\;mod\;2q,\mu \right)=H\left(Ay_{1}+x\;mod\;2q,\mu \right)$$

Equation (4) holds if equation $A_{k}e+x+qz\;mod\;2q=Ay_{1}+x\;mod\;2q$ holds. The detailed steps are given below in Equation (5).

(5) $$\begin{array}{cc} A_{k}e+x+qz\;mod\;2q & =A_{k}\left(y_{1}+{\left(-1\right)}^{t}B_{k}z\right)+x+qz\;mod\;2q\\ \; & =A_{k}y_{1}+{\left(-1\right)}^{t}A_{k}B_{k}z+x+qz\;mod\;2q\\ \; & =A_{k}y_{1}+{\left(-1\right)}^{t}qz+x+qz\;mod\;2q\\ \; & =A_{k}y_{1}+x\;mod\;2q \end{array}$$

### 5.2. Unforgeability

**Theorem** **1.** *This CLRS is unforgeable as the lattice assumption $Z-SIS_{q,n,m,\beta }^{\kappa }$ cannot be solved under the current computation conditions.*

**Proof.**  This section utilizes the method of proof by contradiction to prove the scheme’s unforgeability. According to Definition 6 of the security model, a query–response game was constructed in a random oracle model between an adversary Eve *E* and a challenger Charlie *C*. These two participants execute the following steps to achieve personal goals. *A* wants to obtain more information about the secret key and signature and tries to forge a valid signature of the target message $\mu^{*}$. *C* wants to utilize the forged signature to solve the SIS instance. Here, assume that the successful forgery probability of *E* is ξ. All of these operations should be performed in polynomial time. □

- *Initialize*: *C* initiates the system parameters (n,m,q,k,σ).
- *Query*: *E* performs the following queries with *C* and tries to obtain enough information to increase the probability of forging a legitimate signature.
  – *Partial secret key query*: *E* queries non-target user *i* about partial private key $psk_{i}$. *C* first checks a dedicated list $List_{psk}$ to see if the queried $\left(i,T_{i},S_{i}\right)$ exists or not. If so, *C* returns $\left(T_{i},S_{i}\right)$ to *E*. Otherwise, *C* executes the $TrapGen(1^{n})$ algorithm to derive a new $S_{i}$ and returns it to *E*. Meanwhile, *C* records this partial private key $\left(i,T_{i},S_{i}\right)$ in the list $List_{psk}$. Here, *E* can perform this query with $t_{psk}$ times until obtaining enough information.
  – *Secret key query*: *E* queries non-target user *i* about private key $sk_{i}$. *C* first checks a dedicated list $List_{sk}$ to see if the queried $\left(i,A_{i},B_{i}\right)$ exists or not. If so, *C* returns $\left(A_{i},B_{i}\right)$ to *E*. Otherwise, *C* executes the KeyExt. algorithm to derive a new $B_{i}$ and returns it to *E*. Note that if the partial private key $\left(i,T_{i},S_{i}\right)$ is not queried, *C* must perform the PartialKeyGen. algorithm first and record this result in the list $List_{psk}$. Meanwhile, *C* records this private key $\left(i,A_{i},B_{i}\right)$ in list $List_{sk}$. Here, *E* can perform this query with $t_{sk}$ times until obtaining enough information.
  – *H query*: *E* queries the non-target message μ about the hash algorithm *H*. *C* first checks a dedicated list $List_{H}$ to see if the queried $\left(\mu ,z_{i}\right)$ exists or not. If so, *C* returns $\left(\mu ,z_{i}\right)$ to *E*. Otherwise, *C* executes the first three steps of the Sign algorithm to derive a new hash value $z_{i}$ and returns it to *E*. Meanwhile, *C* records this private key $\left(\mu ,z_{i}\right)$ in list $List_{H}$. Here, *E* can perform this query with $t_{H}$ times until obtaining enough information.
  – *Signature query*: *E* queries the non-target message μ about its signature $s_{\mu }$. *C* first checks a dedicated list $List_{s_{\mu }}$ to see if the queried $\left(\mu ,s_{\mu }\right)$ exists or not. If so, *C* returns $\left(\mu ,s_{\mu }\right)$ to *E*. Otherwise, *C* executes steps 4 and 5 of the Sign algorithm to derive a new signature $s_{\mu }$ and returns it to *E*. Note that if the hash value $\left(\mu ,z_{i}\right)$ and the private key $\left(i,A_{i},B_{i}\right)$ are not queried, *C* must perform the *H* algorithm and KeyExt. algorithm first and record these results in lists $List_{H}$ and $List_{sk}$, respectively. Meanwhile, *C* records this signature $\left(\mu ,s_{\mu }\right)$ in list $List_{s_{\mu }}$. Here, *E* can perform this query with $t_{s_{\mu }}$ times until obtaining enough information.
- *Forge*: With enough queried information, *E* has the ability to forge a secret key $B_{k}^{*}$ and then generate a valid signature $s_{\mu^{*}}^{*}$ of target message $\mu^{*}$.
- *Challenge*: As *C* grasps the users’ public and private keys (pk,sk) from the former query processes, *C* can perform the signing process correctly. Based on the forking lemma, *C* can generate the other one valid signature $s_{\mu^{*}}^{**}$ of the same target message $\mu^{*}$. So, signature $s_{\mu^{*}}^{**}=\left(e^{**},z^{**},x^{**}\right)$ of target message $\mu^{*}$ and the forged signature $s_{\mu^{*}}^{*}=\left(e^{*},z^{*},x^{*}\right)$ satisfy the following Equation (6):
  (6) $$\left\{\begin{array}{cc} z^{*} & =H(A_{k}e^{*}+x^{*}+qz^{*}\;mode\;2q,\mu^{*})\\ \; & =H(A_{k}y_{1}^{*}+x^{*}\;mode\;2q,\mu^{*})\\ z^{**} & =H(A_{k}e^{**}+x^{**}+qz^{**}\;mode\;2q,\mu^{*})\\ \; & =H(A_{k}y_{1}^{**}+x^{**}\;mode\;2q,\mu^{*}) \end{array}\right.$$
- *Analyze*: *C* performs a detailed analysis of Equation (6) and tries to solve the SIS instance. Equation (6) can be changed to Equation (7).
  (7) $$\left\{\begin{array}{c} A_{k}e^{*}+x^{*}+qz^{*}=A_{k}y_{1}^{*}+x^{*}\;mode\;2q\\ A_{k}e^{**}+x^{**}+qz^{**}=A_{k}y_{1}^{**}+x^{**}\;mod\;2q \end{array}\right.$$
  Next, Equation (7) can be changed to Equation (8).
  (8) $$\left\{\begin{array}{c} A_{k}\left(y_{1}^{*}-e^{*}\right)=qz^{*}\;mod\;2q\\ A_{k}\left(y_{2}^{**}-e^{**}\right)=qz^{**}\;mod\;2q \end{array}\right.$$
  Then, we can derive Equation (9) when the first equation is subtracted from the second equation in Equation (8).
  (9) $$A_{k}\left(y_{1}^{*}-e^{*}-y_{2}^{**}+e^{**}\right)=q\left(z^{*}-z^{**}\right)\;mod\;2q$$
  Therefore, we can derive $A_{k}\left(y_{1}^{*}-e^{*}-y_{2}^{**}+e^{**}\right)=0\;mod\;q$ as $z^{*}\neq z^{**}$. According to Definition 4, $v=(y_{1}^{*}-e^{*}-y_{2}^{**}+e^{**})$ is a solution of the SIS instance $A_{k}v=0\;mod\;q$. Now, *C* has successfully solved the hard lattice problem.
  However, the SIS problem cannot be solved with the most advanced computation, so the fact that *C* solved this problem is contrary to fact. So, the former hypothesis that *E* can successfully forge a valid CLRS is invalid, and the proposed CLRS cannot be forged by an adversary. Meanwhile, along with the increased query times, the successful forgery probability ξ of *E* decreases. In the former query–response game, *E* performed $t_{psk}$ partial secret key queries, $t_{sk}$ secret queries, $t_{H}$ hash queries, and $t_{s_{\mu }}$ signature queries, so the probability should be $\frac{\xi }{3/2t_{psk}+3/2t_{sk}+3/2t_{H}+t_{s_{\mu }}}$. Here, 3/2 is due to the assumption that *C* has a 1/2 chance of returning back to the former query. In terms of probability, *E* cannot forge a valid CLRS, and the proposed CLRS scheme is secure.

### 5.3. Signer’s Anonymity

**Theorem** **2.** *The signer in this CLRS scheme is anonymous, as the lattice assumption $Z-SIS_{q,n,m,\beta }^{\kappa }$ cannot be solved with the current computation condition.*

**Proof.**  According to Definition 7, this section also establishes a query–response game between *E* and *C*. Based on the random oracle model, adversary *E* obtains enough information about the CLRS scheme without the target user. The query–response processes are the same as those in the security proof of unforgeability. Then, *C* attempts to distinguish whether the ring user i=0 or i=1 signs the signature. □

- *User creation*: *E* randomly selects two users, $i=0\to (A_{0},B_{0})$ and $i=1\to (A_{1},B_{1})$, and asks the challenger *C* to generate the corresponding signatures.
- *Signature construction*: *C* executes the CLRS scheme and generates a signature by randomly selecting user i(i=0,1). When he selects i=0, he generates signature $\left(e_{0},z_{0},x_{0},\mu \right)$ with keys $\left(A_{0},B_{0}\right)$. When he selects i=1, he generates signature $\left(e_{1},z_{1},x_{1},\mu \right)$ with keys $\left(A_{1},B_{1}\right)$. Then, he sends the generated signature to *E*.
- *Guess*: When *E* receives signature $\left(e_{i},z_{i},x_{i},\mu \right)$, *E* performs a guess i=0 or i=1. Next, *C* publishes the correct results. Here, *E* presents correct results with a probability of 1/2.
- *Analysis*: First and foremost, this signature is constructed with the lattice assumption $Z-SIS_{q,n,m,\beta }^{\kappa }$. *E* cannot solve this lattice hard problem and cannot obtain any information about the keys $\left(A_{0},B_{0}\right)$ (or $\left(A_{1},B_{1}\right)$) that *C* selects for signing. Secondly, the parameter $\left(y_{1},y_{2}\right)$ is chosen with a bimodal Gaussian distribution $D_{\sigma }^{m}$, and this distribution is uniform. So, every selection is different, and *E* cannot obtain any information from other former generated signatures. Then, the bit t∈{0,1} in the signing process is selected randomly, which also guarantees the uncertainty of the signature at every signing time. Therefore, the statistical distance between two signatures $\left(e_{0},z_{0},x_{0},\mu \right)$ and $\left(e_{1},z_{1},x_{1},\mu \right)$ is also indistinguishable, and the probability of success is 1/2 for *E* each time.

Here, this CLRS scheme can achieve anonymity as the other users cannot distinguish the real signer of the ring from the signature. Meanwhile, to guarantee traceability, it can confirm the real signer with the **Verify** algorithm when some disputes occur.

Now, the proposed CLRS scheme has been proven to have unforgeability and anonymity security properties.

### 5.4. Other Security Properties

The former two properties guarantee basic security. This CLRS scheme can also achieve the following security properties:

*Non-repudiation*: For one data transaction in the IoT network, it contains all the signatures of the related operators. When the signature is open, the signer cannot deny their signature as it is signed with their own private key.

*Traceability*: IoT data are transmitted among different IoT devices and systems frequently, so data traceability is essential for data loss and privacy leakage. This CLRS scheme can guarantee data traceability as every process is signed by the corresponding operator. When some disputes occur, it can trace the related operators with this signature.

*Post-quantum security*: In the coming quantum computer age, the current information systems equipped with traditional cryptographic algorithms are not secure. This CLRS scheme is constructed with the post-quantum lattice hard problem $Z-SIS_{q,n,m,\beta }^{\kappa }$ which guarantees safety against anti-quantum attacks.

*Resist malicious KGC*: In some information systems, a malicious KGC leads to the serious destruction of system security and user privacy. This CLRS scheme can resist a malicious KGC as the signature secret key $A_{i}$ is generated with two parts: one partial secret key $T_{i}$ generated by the KGC and one secret matrix $K_{i}$ selected by the users themselves. The malicious KGC cannot impersonate the signer to sign as they do not know the secret matrix $K_{i}$. This mechanism guarantees users’ secret key security once the KGC becomes malicious.

## 6. Efficiency Comparison

This section compares the efficiency of the CLRS scheme from two aspects: key size and time consumption. Here, two related CLRS schemes in [20,21] were selected for comparison.

### 6.1. Key Size Comparison

**Figure 1:**
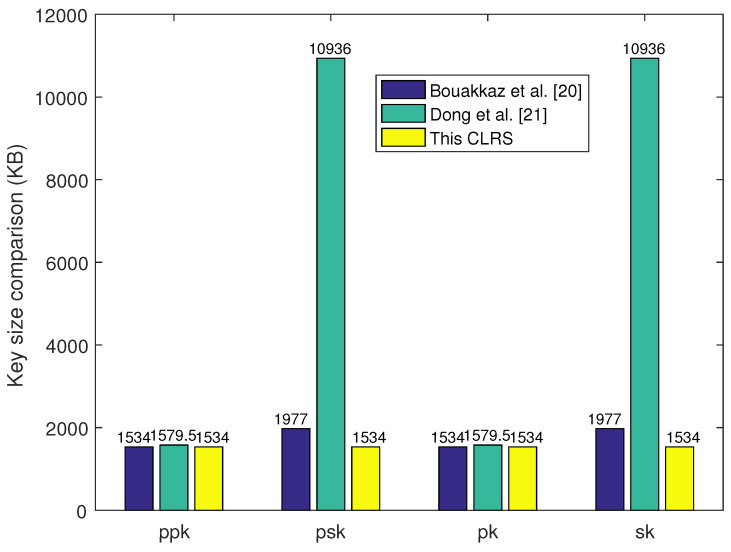
Key size comparison.

**Figure 2:**
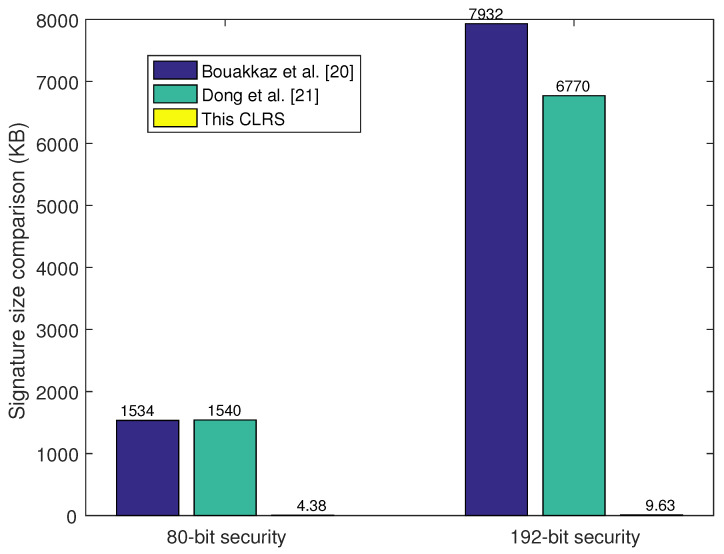
Signature size comparison.

Key size affects the system storage and program implementation efficiency, so a smaller key size leads to a more efficient CLRS scheme. The key size comparison results are shown in Table 2 as well as Figure 1 and Figure 2. In Ref. [21], the key sizes of ppk, psk, pk, and sk are a little bigger than those in the proposed CLRS scheme as the parameter *k* is set at k>n. From the results of the theoretical analyses, the key size in the proposed CLRS is smaller than that in the other two CLRS schemes. Meanwhile, the system parameters were set and executed on a Windows 11 laptop with Intel Core i7-9700 CPU 3.0 GHz and 16 GB RAM. Here, two security levels were selected according to the setting principle in [24]; the 80-bit (192-bit) security level was equal to a 512-bit (1024-bit) security level in a traditional cryptographic algorithm. The 80-bit security level was defined with $q=2^{23}$, n=512, m=3545, and $\sigma =2^{30}$, and the 192-bit security level was defined with $q=2^{27}$, n=1024, m=7807, and $\sigma =2^{30}$. Here, parameter *k* was set at k=3650 and k=7900 for 80-bit and 192-bit security levels, respectively. With these parameters, the key size of these three schemes was computed, and the comparison of the results is shown in Table 3. These results are also shown in Figure 1 and Figure 2. These two figures visually show the results of the proposed CLRS scheme compared with the two similar CLRS schemes in terms of key and signature size. The key sizes of psk and sk in the proposed CLRS are much smaller than those in Ref. [21], and the signature size is much smaller than that in the other two CLRS schemes.

Although the key size seems a little bigger than the current ECC and RSA cryptographic algorithms, it can guarantee post-quantum security for IoT networks. Meanwhile, the popularization of quantum algorithms can continuously improve the computational efficiency of PQC so PQC is more suitable for applications in IoT networks. It also can utilize the pre-generate method to prepare the keys to reduce scheme performance time. Moreover, this CLRS, with its very small size, will be very suitable for IoT data transmission through different IoT devices.

### 6.2. Time Consumption Comparison

**Figure 3:**
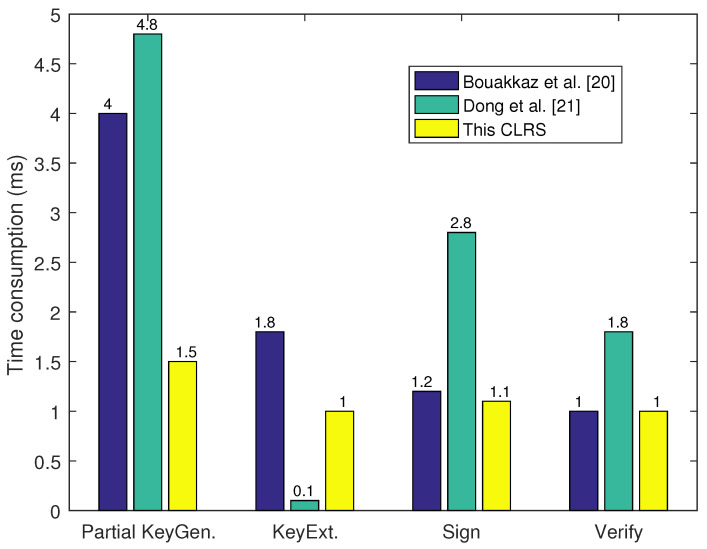
Time consumption.

For the time consumption of the proposed CLRS scheme, this section selects some essential operations, such as trapdoor, Gaussian sample, matrix multiplication, and hash, for evaluations. Here, $T_{Trap}$ represents the TrapGen algorithm, $T_{Samp}$ represents the reject sample algorithm, $T_{Mul}$ represents the matrix multiplication, and $T_{H}$ represents the hash algorithm. With the former performance environment, this CLRS scheme was executed 10 times, and the time consumption of each algorithm was averaged over the test. $T_{Trap}$, $T_{Samp}$, $T_{Mul}$, and $T_{H}$ required 1.5 ms, 1 ms, 0.1 ms, and 0.8 ms. The comparison of the results is shown in Table 4 and Table 5 as well as Figure 3. As shown in the table, the proposed CLRS scheme has fewer operations than the other two CLRS schemes. Meanwhile, under the former experiment environment, this time consumption was determined with the same system parameters. Figure 3 visually shows the results of the proposed CLRS scheme compared with two similar CLRS schemes in terms of every signature step.

## 7. Example Application

**Figure 4:**
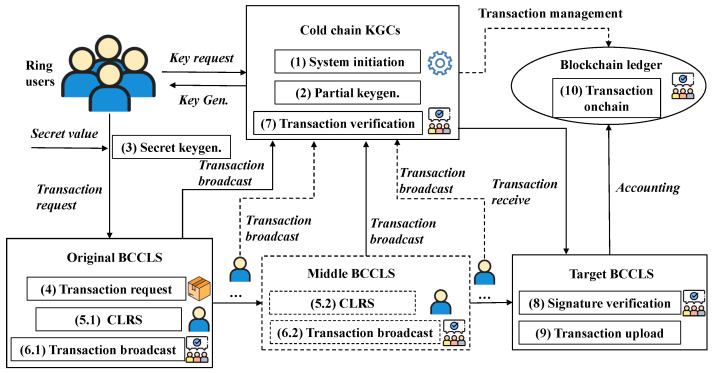
Example application in BCCLS.

Using this CLRS scheme can improve the security during data sharing among different IoT devices. This section presents an example of the logistics data-sharing transaction in a blockchain-based cold-chain logistics system (BCCLS), which is simply shown in Figure 4. To guarantee the security of cold-chain goods, all the logistics processes need to be monitored, and all the operations need to be recorded. This logistics data-sharing process mainly contains 10 steps, which are shown in the following.

1. *System initiation*: The cold-chain KGCs in different BCCLSs compose a union and establish a distributed logistics data-sharing platform as the BCCLS network. Every KGC initiates the BCCLS and derives the system parameters pp. This step mainly establishes a CLRS framework and prepares for the signature algorithm.
2. *Partial keygen.*: The KGC generates partial public and secret keys $\left(ppk_{i},psk_{i}\right)$ for the system user. These partial keys guarantee that users perform the signature according to the principle of the CLRS scheme. Meanwhile, they can enable the authentication of user identity as the user cannot deny the signature with these partial keys.
3. *Seret keygen.*: The user selects a secret value and composes it with the partial keys to generate their own public and secret keys $\left(pk_{i},sk_{i}\right)$. This mechanism can resist a malicious KGC as the KGC does not grasp the full keys.
4. *Transaction request*: Once a cold-chain good needs to be transported, the original BCCLS must initiate a blockchain transaction with the target BCCLS first. Then, all the related transaction processes are recorded in this transaction. The related operators should sign this transaction according to the signing steps of the CLRS scheme.
5. *CLRS*: The step “5.1, 5.2, ⋯" represents the singing processes in different CCLSs. When the cold-chain goods are transported from the producing area to a supermarket, the data exchange in different parts should be recorded in the central server. Meanwhile, the related operators sign this transaction with their own keys $\left(pk_{i},sk_{i}\right)$. Note that one operator serves as the ring member in one BCCLS, which can represent the ring to sign this transaction. This ring signature mechanism can guarantee the signer’s anonymity.
6. *Transaction broadcast*: The signed transactions should be broadcasted to the BCCLS network. This process mainly guarantees transaction validity and network-wide consistency.
7. *Transaction verification*: The cold-chain KGC union takes responsibility for transaction verification. This process is similar to the transaction in the Bitcoin system: all the transactions should be selected, verified, and packaged. Every KGC verifies the legality of these transactions, signs with its private key, and returns the verification results back to the union manager. Only the valid transactions are packaged and served as the newest block of the blockchain ledger.
8. *Signature verification*: When the target BCCLS receives the transaction, the signature validity is verified. With signature (e,z,x) and message μ, the verifier utilizes the signer’s public key to perform this verification. Only through this verification can the transaction be accepted by the target user.
9. *Transaction upload*: The transactions are uploaded to the BCCLS network only by passing the former verification step. The cold-chain KGC union performs this verification process and utilizes the consensus protocol to achieve network consistency.
10. *Transaction onchain*: All the valid transactions are recorded in the unified blockchain ledger in the BCCLS network. Meanwhile, these transactions are also recorded in the corresponding BCCLS of the transaction initiator and receiver. When these transactions are recorded in the blockchain ledger, they become immutable records. This operation not only protects data and privacy but also establishes a traceability mechanism for process safety.

**Figure 5:**
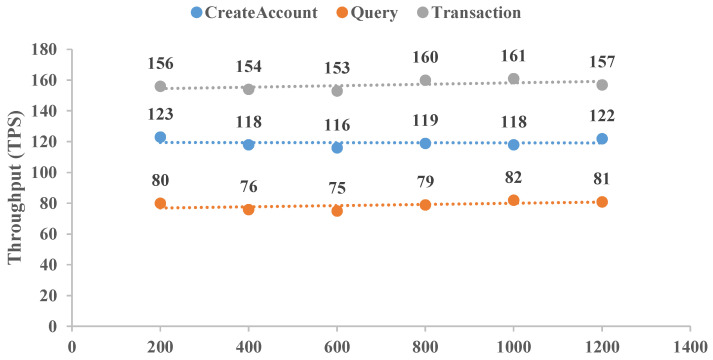
Transaction throughput comparison.

**Figure 6:**
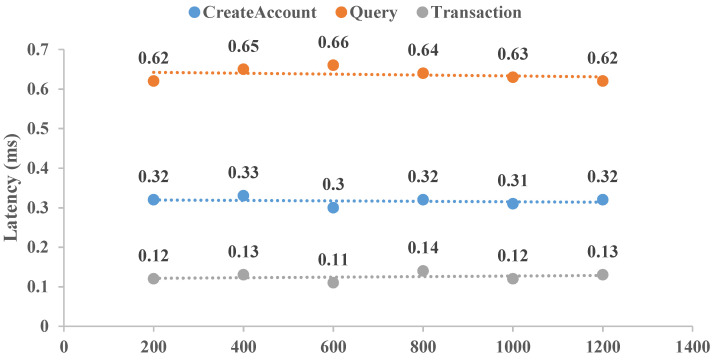
Transaction latency comparison.

To reflect the application of this CLRS scheme, a distributed BCCLS with five nodes was established with Hyperledger Fabric. Here, two items of transaction throughput and latency were selected and executed. The throughputs of “CreatAccount”, “Query”, and “Transaction” were determined 10 times, and the averaged results are shown in Figure 5. Meanwhile, the performance results of transaction latency for these same aspects are shown in Figure 6. “CreatAccount” represents the generation rate of user registration, “Query” represents the number of queries of cross-chain transaction origination, and “Transaction” represents the establishment of the transaction amounts. These results show that the BCCLS system equipped with this CLRS scheme can remain stable with increasing transaction numbers. For these three aspects, “Query” is executed with high throughput by the low transaction latency, and “Transaction” is executed with low throughput by the high transaction latency. Moreover, cold-chain logistics data are essential for commodity safety, route optimization, and scientific research. Studying post-quantum security cryptographic algorithms is necessary to think ahead about IoT network security in the age of quantum computers. Meanwhile, the proposed CLRS scheme is also fit for other data-sharing needs in finance, the Internet of Vehicles, and the Internet of Medical Things.

## 8. Conclusions

This paper proposes a CLRS scheme based on a lattice hard problem SIS. In this scheme, a certificateless mechanism is applied to weaken the KGC, which can reduce the security risk posed by a malicious KGC. Meanwhile, a ring signature mechanism is applied to protect the signer’s privacy, which hides the real signer in the ring to achieve signer anonymity. Then, the security proofs proved that the proposed CLRS scheme is correct and unforgeable, ensuring the signer’s anonymity with non-repudiation, traceability, and post-quantum security. Moreover, the comparison results of efficiency, time, and energy consumption show that the proposed CLRS scheme is very efficient and practical. This scheme improves the post-quantum security of IoTs network and is better suited for protecting data-sharing communication among different IoT devices.

In future work, two work aspects should be considered. One is the attribute-based cryptographic algorithm, which can integrate the attributes of the IoT into the scheme to achieve the flexible access control of IoT devices. The other one is the application of these PQC algorithms in IoT networks to explore the efficient practical application of these algorithms.

## Figures and Tables

**Table 1:** Parameter definitions.

Notation	Meaning
n	Security parameter
q	A prime number
m	A positive integer with m≥2n⌈logq⌉
β	Error parameter
*L*	Threshold parameter
σ	Standard deviation
$D_{\sigma }^{m}$	Bimodal Gaussian distribution
$H_{1},H_{2}$	Cryptographic Hash function
μ	Message

**Table 2:** Key size comparison.

Scheme	ppk	psk	pk	sk	sig.
Bouakkaz et al. [20]	mnlogq	$(2n^{2}+mn)\;logq$	mnlogq	$(2n^{2}+mn)\;logq$	mnlogq
Dong et al. [21]	nklogq	mklogq	nklogq	mnlogq+mklogq	(2m+mn)logq
Proposed CLRS	mnlogq	mnlogq	mnlogq	mnlogq	mlog(12σ)

**Table 3:** Key size comparison results (KB).

Scheme	ppk	psk	pk	sk	sig.
Bouakkaz et al. [20]	1534	1977	1534	1977	1534
Dong et al. [21]	1579.5	10,936	1579.5	10,936	6770
Proposed CLRS	1534	1534	1534	1534	9.63

**Table 4:** Time cost comparison.

Scheme	Partial KeyGen.	KeyExt.	Sign	Verify
Bouakkaz et al. [20]	$2T_{Trap}+T_{Samp}$	$2T_{Mul}+2T_{H}$	$4T_{Mul}+T_{H}$	$2T_{Mul}+T_{H}$
Dong et al. [21]	$2T_{Trap}+T_{Samp}+T_{H}$	$T_{Mul}$	$4T_{Mul}+3T_{H}$	$2T_{Mul}+2T_{H}$
This CLRS	$T_{Trap}$	$2T_{Mul}+T_{H}$	$3T_{Mul}+T_{H}$	$2T_{Mul}+T_{H}$

**Table 5:** Time cost comparison results (ms).

Scheme	Partial KeyGen.	KeyExt.	Sign	Verify
Bouakkaz et al. [20]	4	1.8	1.2	1
Dong et al. [21]	4.8	0.1	2.8	1.8
This CLRS	1.5	1	1.1	1

## Data Availability

The original contributions presented in this study are included in this article. Further inquiries can be directed to the corresponding author.
